# Diabetes Might Augment the Severity of COVID-19: A Current Prospects

**DOI:** 10.3389/fcvm.2020.613255

**Published:** 2021-01-05

**Authors:** Jayeeta Sur, Juhi Sharma, Divakar Sharma

**Affiliations:** ^1^Independent Researcher, Kolkata, India; ^2^Department of Botany and Microbiology, St. Aloysius College, Jabalpur, India; ^3^Central Research Facility (CRF), Mass Spectrometry Laboratory, Kusuma School of Biological Sciences, Indian Institute of Technology Delhi, New Delhi, India

**Keywords:** diabetes, COVID-19, SARS-CoV-2, hyperglyceamia, ACE2

## Introduction

COVID-19 is the recently emerged outbreak across the globe which is caused by SARS-CoV-2 (enveloped, positive single-stranded RNA virus). To date, more than 49.7 million confirmed cases and more than 1.2 million deaths have been reported worldwide (10 November 2020), making COVID-19 a major human health concern (1). Predominantly, SARS-CoV-2 infects lower airways and creates respiratory as well as systemic illness which further progresses to a severe form of pneumonia-like conditions in 10–15% of patients (2). SARS-CoV-2 can infect people of any age, but old-age people as well as people with comorbidities like chronic obstructive pulmonary disease (COPD), cerebrovascular disease, coronary heart disease, hypertension, tuberculosis, tobacco smoking, and diabetes are under a higher risk to get SARS-CoV-2 infection and develop COVID-19. Various reports suggested that these comorbidities might potentiate the COVID-19 progression and severities (3–5); therefore, researchers get attention toward this direction and try to manage this situation.

Various studies revealed that among the cases of COVID-19 with comorbidities, 12–22% cases have diabetes, and the mortality rate in these cases is 6–7% (3–6). In this opinion, we have to provide a summary of the symptomatic characteristics of COVID-19 with diabetes, and critical assessment of the association between COVID-19 and diabetes. Therefore, this opinion article could provide meaningful insight for future research and contribute to better clinical management of diabetes patients with COVID-19.

## Could Diabetes Potentiate COVID-19

Recently, a report by Yang and colleagues has shown (4) that out of a group of 52 ICU patients with COVID-19, the distinct comorbidities of 32 non-survivors were cerebrovascular diseases (22%, 7 cases) and diabetes (22%, 7 cases). Similarly, a study of 1,099 patients with confirmed COVID-19 showed that out of 1,099 cases, 173 were the case of comorbidities of which 16.2% were diabetic (3). A study also showed that out of 140 patients admitted being COVID-19 positive, 12% of patients had diabetes (5). These studies have shown that COVID-19 patients with diabetes are treated with angiotensin-converting enzyme (ACE) inhibitors, but their treatments were not assessed. China's largest epidemiological investigation also showed COVID-19 mortality with diabetes up to 7.3% (80/1,102), which is dramatically higher than that of patients without any comorbidity (0.9%, 133/15,536) (7). Kulscar et al., through an *in vivo* experiment using type 2 diabetic transgenic mouse models that express the DDP-4 receptor on pulmonary alveolar cells, have studied the effect of diabetes on MERS-coronavirus infection and its severity. They observed a remarkable association of diabetes with greater weight loss and pulmonary inflammation having macrophage infiltration as similar to the clinical disease (8).

The most striking observation is that the mortality rate in men is higher than in women diabetic patients who were infected by SARS-CoV-2 (4). Men are more affected by COVID-19 as compared to women, which are probably due to three times higher expression of ACE2 in men than women (9). In an experiment, the continuous infusion of angiotensin 1–7 has shown a vasodilator effect only in female rats (10). The main reason behind this observation is that women are less severely affected by COVID-19 than men which might be due to the protective effect of estrogen-mediated ACE2 regulation in premenopausal women (11). A study showed the potential influence of menstrual status and sex hormones on females infected with SARS-CoV-2 and suggested menopause, an independent risk factor for female COVID-19 patients. Estradiol (E2) is a protective factor and regulates the cytokine-related immune responses in premenopausal women (12). Therefore, post-menopausal women lose the protective effects of estrogens so they have a potential risk for type-2 diabetes; hence, this comorbidity definitely increases the risk for COVID-19. Recommended drugs for diabetes and associated comorbidities like pioglitazone, glucagon-like peptide-1 agonists, statins, diuretics, and mineralocorticoid inhibitors increase the expression of ACE2 level (13). It has been shown that angiotensin-converting enzyme inhibitors (ACEIs) and angiotensin-receptor blockers (ARBs) increase the ACE2 expression level in the brain, heart, and kidney (11).

## Role of ACE/ACE2 in Diabetes and COVID-19

The angiotensin-converting enzyme (ACE) is secreted in the lungs and kidneys by the endothelium (inner layer) of blood vessels that regulate the volume of the fluids in the body and hence control blood pressure. ACE is the main component of the renin–angiotensin system (RAS) and converts angiotensin-I (ANGI) to the active angiotensin-II (ANGII). ANGII further increases blood pressure (BP) and inflammation, which causes tissue injury as well as increases damage to blood vessel linings. Angiotensin-converting enzyme 2 (ACE2) is also an important element of the RAS pathway which counteracts the effects of ANGII. It is reported that ACE2 expressed by epithelial cells of the lung, kidney, intestine, and blood vessels helps pathogenic coronaviruses such as SARS-CoV and SARS-CoV-2 in their target cell binding and endocytosis (14).

ACE2 is considered as the host cell surface receptor that interacts directly with the spike glycoprotein (S protein) of SARS-CoV-2 (3). A recent study has been suggested that ACE2 has 10–20 times higher binding affinity to the receptor-binding domain (RBD) of SARS-CoV-2 than SARS-CoV (15). Spike proteins of SARS-CoV-2 bind to host ACE2, so theoretically the normal functioning of ACE2 is imbalanced which makes the aggravation of ANGII effect, which may further cause inflammation, death of alveoli cells, and tissue injury especially to the heart and lungs in COVID-19 patients.

After consideration of the facts that the utilization of ACE2 binds the spike of SARS-CoV-2 and after further endocytosis (16, 17), it led to the reduction of ACE2 availability which resulted in the overactivation of RAS (18). Therefore, this overactivation of RAS could be responsible for the adverse risk of COVID-19 in patients with diabetes. In this regard, the application of RAS inhibitors in patients with COVID-19 with pre-existing diabetes could be a potential therapeutic option.

It is evident from a study that entry of SARS-CoV-2 to the host cell depends on spike-ACE2 interaction and this can be blocked by transmembrane serine protease-2 (TMPRSS2) inhibitors because TMPRSS2 has been used for spike (S) protein priming by SARS-CoV-2 (19). It also indicates that the antibodies against SARS-CoV could partially protect against SARS-CoV-2 infection. These outcomes have significant implications to understanding SARS-CoV-2 transmissibility and pathogenesis and reveal therapeutic intervention targets.

A significant increase of the ACE2 expression in type 1 or type 2 diabetic patients has been reported while they are treated with ACEIs and ARBs (14). This report suggested that patients with diabetes were treated with drugs that increased the ACE2 expression, so such patients could be at a higher risk of SARS-CoV-2 infection and severity of COVID-19. However, other meta-analysis suggested no significant association between the use of ACEIs or ARBs with the risk of SARS-CoV-2 infection and severity of COVID-19 in hypertensive patients and other old-age patients (6) which is just opposite to COVID-19 with diabetes comorbidities (14). Still, more research is needed to support these contradictory facts. Therefore, these COVID-19 patients with comorbidities should be monitored for ACE2-modulating medications, like ACEIs and ARBs (20).

At low intracellular pH, SARS-CoV-2 spikes bind to the host ACE2 enzyme and enter into the cell, causing the infection (21, 22). Diabetes may have a strong association with low intracellular pH and could potentiate the SARS-CoV-2 infection. SARS-CoV-2, ACEIs, and ARBs bind to ACE2 which can further increase the level of ANGII. The increased level of ANGII can activate the Na^+^/H^+^ exchanger (NHE) which further reduces the intracellular pH. NHE pumps 3 Na^+^ ions into the cell and 2 H^+^ ions out of the cell (21). Apart from that, it also transports Ca^2+^ ions inward the cell. Overall transports of ions make the intracellular environment acidic and hypoxic, which leads to the production of reactive oxygen species (ROS) and cell death. NHA2 is the isoform of the Na^+^/H^+^ exchanger (NHE) present in the beta cells of the pancreas and involved in the secretion of insulin (23). In diabetic patients, continuous activation of NHA2 through SARS-CoV-2 infection and a higher level of ANGII lead to pancreatic beta-cell injury via the overproduction of ROS. Therefore, sustainable activation of NHA2 in COVID-19 and diabetes coinfection can damage the pancreatic beta cells or tissue of the pancreas permanently and lead to disease severity as well as death. Consequently, the use of ACEIs and ARBs in diabetes patients can lead to increased morbidity and COVID-19 mortality.

## Cytokine Storm and Biomarker Level in Diabetic Patients Due to COVID-19

Inflammatory cytokine secretion may occur due to immune system imbalance. The cytokine is released from white blood cells and transmits signals from one immune cell to another especially during the entry of unwanted invaders. When cytokines are released in large volume due to hyperactivity of the immune cells, this phenomenon is called a cytokine storm (24). Higher levels of pro-inflammatory cytokines are also observed in COVID-19 patients (25). Data from Chinese studies showed that diabetic patients with COVID-19 displayed higher IL-6 as well as C-reactive protein and cause severe pulmonary infection and death. Cytokine storm is the major key factor for this pulmonary infection and death (3–5, 26). In diabetes patients, hyperglycemia stimulates the synthesis of cytokines. Some inflammation-related biomarkers like IL-6, serum ferritin, ESR, and CRP are present in higher concentrations in diabetic patients and linked to the disease-related cytokine storm. Among them, IL-6 is a very good predictor for disease prognosis and severity because its expression time is longer than other cytokines such as TNF and IL-1 (3, 5). In addition to this, a significant amount in serum ferritin indicates the activation of the monocyte–macrophage system, which is crucial for an inflammatory storm (27). These results indicate that patients with diabetes-COVID-19 coinfection are more susceptible to formation of an inflammatory storm, which eventually leads to rapid deterioration of COVID-19.

A study showed that urea nitrogen, albumin, and N-terminal probrain natriuretic peptide (NT-proBNP) level increased in diabetes with cardiovascular diseases (28). Diabetes and other comorbidities may have a possible association to COVID-19 infection which showed progressive systemic injury in diabetic patients (6, 14).

## Hyperglycemia: A Potential Risk Factor

**Figure 1:**
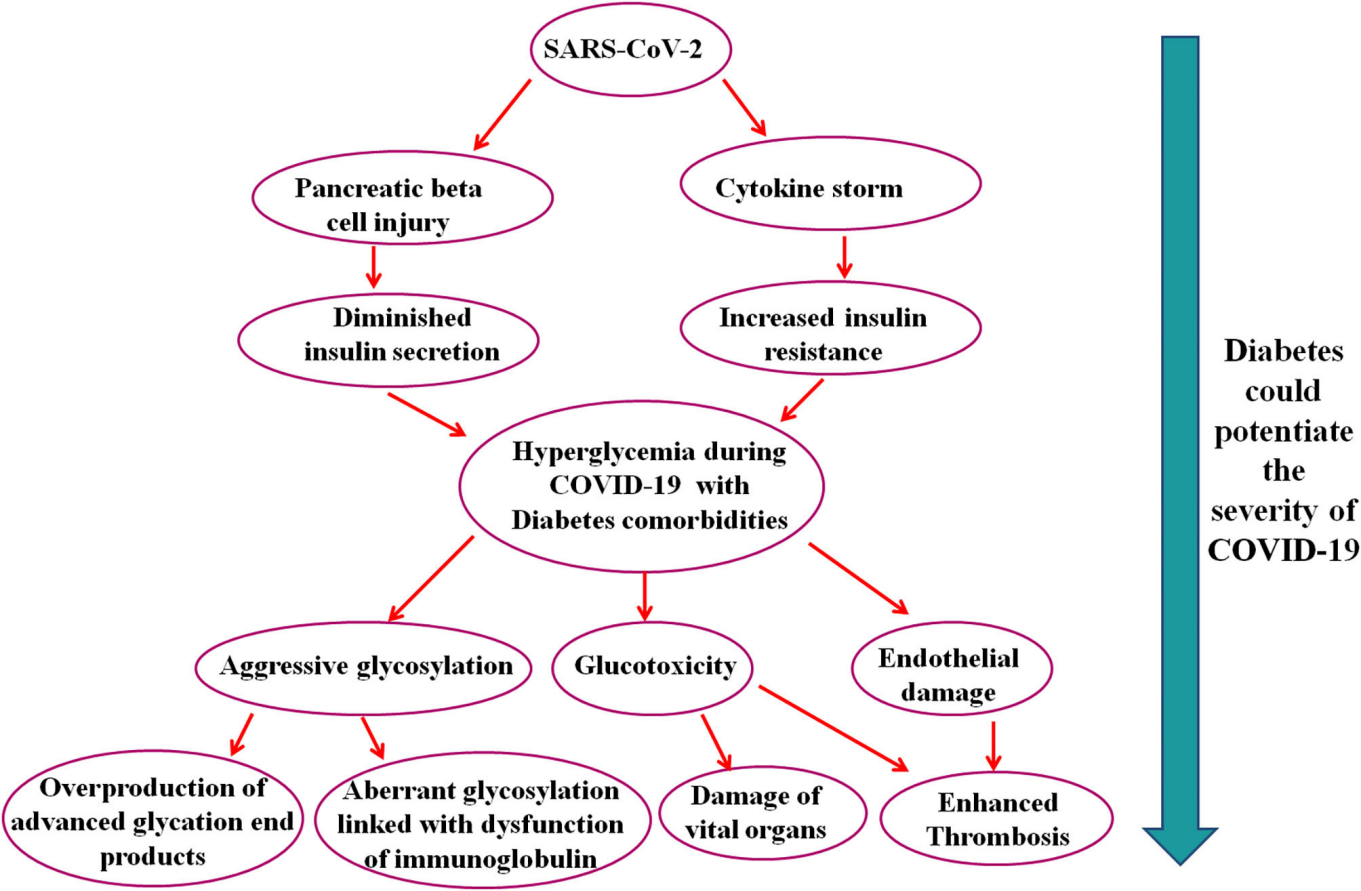
Schematic representation of the possible pathways that suggest that diabetes could potentiate the severity of COVID-19.

Diabetes crucially potentiates COVID-19 development and adverse endpoints; thereby, well-controlled blood glucose, maintaining glycemic variation (range 3.9–10.0 mmol/L), is associated with substantial decreases in adverse composite outcomes and death (29). Hyperglycemia is the potential risk factor for mortality and severity of SARS related diseases (30). It has been reported that hyperglycemia induced by diabetes linked to aggressive glycosylation and led to overproduction of advanced glycation end products (31). A study showed that aberrant glycosylation was associated with the dysfunction of immunoglobulin (32). Diabetes and SARS-CoV-2 coinfections can trigger stress and increase the secretion of hyperglycemia hormones, such as glucocorticoid and catecholamines, which results in increased blood glucose concentration and other diabetic complications (7). In Figure 1, we have summarized the potential mechanisms that explain the severity of COVID-19 in patients with diabetes comorbidity.

In brief, this manuscript shows some clue about SARS-CoV-2 infection in patients with diabetes comorbidity and further suggests that diabetes could potentiate the severity of COVID-19 through various pathways which ultimately lead to advanced glycation end products, glucotoxicity, endothelial damage, vital organ damage, and death. These pathways might open a new avenue for future research and could employ to manage COVID-19 with diabetes comorbidity.

## Author Contributions

DS designed the concept. JSu and JSh drafted the manuscript. DS edited, finalized, and polished the manuscript. All authors approved the final manuscript.

## Conflict of Interest

The authors declare that the research was conducted in the absence of any commercial or financial relationships that could be construed as a potential conflict of interest.
